# Magnesium Potassium Phosphate Compound for Immobilization of Radioactive Waste Containing Actinide and Rare Earth Elements

**DOI:** 10.3390/ma11060976

**Published:** 2018-06-08

**Authors:** Sergey E. Vinokurov, Svetlana A. Kulikova, Boris F. Myasoedov

**Affiliations:** Vernadsky Institute of Geochemistry and Analytical Chemistry, Russian Academy of Sciences, 19 Kosygin st., Moscow 119991, Russia; kulikova.sveta92@mail.ru (S.A.K.); bf@geokhi.ru (B.F.M.)

**Keywords:** magnesium potassium phosphate compound, actinides, rare earth elements, uranium, plutonium, americium, lanthanum, neodymium, immobilization, leaching

## Abstract

The problem of effective immobilization of liquid radioactive waste (LRW) is key to the successful development of nuclear energy. The possibility of using the magnesium potassium phosphate (MKP) compound for LRW immobilization on the example of nitric acid solutions containing actinides and rare earth elements (REE), including high level waste (HLW) surrogate solution, is considered in the research work. Under the study of phase composition and structure of the MKP compounds that is obtained by the XRD and SEM methods, it was established that the compounds are composed of crystalline phases—analogues of natural phosphate minerals (struvite, metaankoleite). The hydrolytic stability of the compounds was determined according to the semi-dynamic test GOST R 52126-2003. Low leaching rates of radionuclides from the compound are established, including a differential leaching rate of ^239^Pu and ^241^Am—3.5 × 10^−7^ and 5.3 × 10^−7^ g/(cm^2^∙day). As a result of the research work, it was concluded that the MKP compound is promising for LRW immobilization and can become an alternative material combining the advantages of easy implementation of the technology, like cementation and the high physical and chemical stability corresponding to a glass-like compound.

## 1. Introduction

Long-term controlled storage or disposal is one of the key stages of the liquid radioactive waste (LRW) management in terms of radiation safety. The preparation of the LRW for this stage involves the transfer of waste into a stable solidified form using preserving matrices [1,2]. Cementation has found wide use in the nuclear industry for radioactive waste (RW) management of low and intermediate activity levels, in spite of significant disadvantages of the method, especially the relatively low degree of incorporation of waste salts, as well as low hydrolytic stability and frost resistance of cement compound. Vitrification is currently the only high level waste (HLW) management technology that is applied in industry [3]. The disadvantages of the method are low chemical and crystallization resistance of the glass at elevated temperatures, as well as the need to use expensive high temperature melters, the liquidation of which, after the end of a relatively short technical lifetime, represents an unresolved radioecological problem.

Ceramic materials [4], and especially synthetic analogues of natural phosphate minerals [5,6], are considered as an alternative to cement and glass for the immobilization of RW, primarily obtained after the reprocessing of spent nuclear fuel (SNF) and containing long-lived isotopes of highly toxic actinides and rare earth elements (REE).

The mineral-like phosphate materials that were obtained at room temperature in aqueous solution by chemical interaction, as a rule, between metal (II) oxides (MgO, ZnO, FeO, CaO) and orthophosphoric acid (H_3_PO_4_) or its derivatives (for example, (di) hydrogenphosphates of metals (I) or ammonium) [7,8] are of particular interest.

Previously, we and other researchers demonstrated [9,10,11,12,13,14,15,16] that magnesium potassium phosphate (MKP) compound based on the MgKPO_4_ × 6H_2_O matrix obtained as a result of the reaction (1), which is an analog of the natural mineral K-struvite [17], is a promising low-temperature material for the immobilization of various RW types.

MgO + KH_2_PO_4_ + 5H_2_O → MgKPO_4_ × 6H_2_O (1)

This method of RW management combines versatility, equipment simplicity and economic efficiency similar cementation, and the obtained MKP compound has a high physical and chemical stability.

The practical use possibility of MKP compound in RW management has to be explained in the context of reliability under the long storage of hazardous RW components mainly highly toxic plutonium and minor actinides, as well as REE, whose content is about half the content of all the metals in HLW. It should also be noted that, although uranium is maximally recovered from solutions during SNF reprocessing for its reuse in the fuel cycle, the residual uranium content (including isotopes U-232, 235, 236, 238) in HLW is about 3 g·L^−1^. Thus, information on the behavior of uranium during immobilization in the MKP compound also has scientific interest.

The data on the phase composition, structure, and hydrolytic stability of synthesized MKP compounds containing uranium, plutonium, americium, and REE (on the example, lanthanum and neodymium) are presented in this article.

## 2. Materials and Methods

The experiments were performed in the glove box (PERERABOTKA, Dzerzhinsk, Russia). The chemicals used in the experiments were of no less than chemically pure grade. Samples of MKP compounds were prepared, according to the procedure previously given in reference [10]. For study, the forms of location and behavior during leaching of uranium and REE by the example of lanthanum in the MKP compound, concentrated aqueous solutions of their nitrates with a metal concentration 228.3 and 242.4 g·L^−1^, respectively, were solidified.

The hydrolytic stability of MKP compound to the leaching of actinides and neodymium as a simulator of the REE group was carried out after the solidification of the HLW surrogate solution of 1000 MW water-water energetic reactor (WWER-1000). The HLW surrogate solution was prepared by dissolving the metal nitrates in an aqueous solution of nitric acid, molybdenum was added in the form of MoO_3_ (Table 1). Preparation of the surrogate solution to solidification was carried out by neutralizing it to pH 8.0 ± 0.1 with sodium hydroxide solution at concentration 15.0 ± 0.1 mol·L^−1^.

The phase composition of the prepared MKP compounds was determined by X-ray diffraction (XRD) (Ultima-IV, Rigaku, Tokyo, Japan). The X-ray diffraction data were interpreted using the specialized Jade 6.5 program package (MDI, Livermore, CA, USA) with PDF-2 powder database.

The structure of samples containing uranium and lanthanum was studied by the scanning electron microscopy (SEM) using microscopes Jeol JSM-6480LV (JEOL, Tokyo, Japan) and LEO Supra 50 VP (LEO Carl Zeiss SMT Ltd, Oberkochen, Germany), respectively. The electron probe microanalysis of the samples was performed using an energy-dispersive analyzer X-MAX 80 (Oxford Instruments plc, Abingdon, England).

The hydrolytic stability of compounds was determined using the semidynamic test, in accordance with GOST R 52126-2003 [18]. Conditions: Monolithic compound 2 cm × 2 cm × 2 cm; leaching agent—bidistilled water (pH 6.6 ± 0.1, volume 200 mL), temperature 23 ± 2 °C, periodic replacement of the leaching agent after 1, 3, 7, 10, 14, and 21 days, the total duration of the test was limited to 28 days. The content of lanthanum, neodymium, and uranium in solutions after leaching was determined by inductively coupled plasma atomic emission spectrometry (ICP-AES) (iCAP-6500 Duo, Thermo Scientific, Waltham, MA, USA), inductively coupled plasma mass spectrometry (ICP-MS) (X Series2, Thermo Scientific, Waltham, MA, USA), and by spectrophotometry (Cary 100 Scan, Varian, Palo Alto, CA, USA), and the content of ^239^Pu and ^241^Am—radiometric method with using of the α-spectrometer (Alpha Analyst, Canberra, Australia).

The mechanism of leaching of the compound components (lanthanum, neodymium, uranium, plutonium, and americium) from the samples was evaluated according to the model [19], described by the linear relationship of log (B_i_) from log (t), where B_i_ is the total yield of the element from the compound during contact with water, mg·m^−2^; t is the contact time, days. The calculate procedure of B_i_ is given in [9,20]. The following mechanisms of element leaching from the compound correspond to various values of the slope in this equation: >0.65—surface dissolution; 0.35–0.65—diffusion transport; <0.35—surface wash off (or a depletion if it is found in the middle or at the end of the test) [9,10,20,21,22,23].

## 3. Results

As a result of the performed experiments, the samples of MKP compounds with a density of 1.75 ± 0.07 g·cm^−3^ were prepared. The content of metals in compounds that were obtained under solidification of uranium and lanthanum nitrate solutions was 6.2 wt % uranium (hereinafter, compound #1) and 6.7 wt % lanthanum (hereinafter, compound #2), respectively. The salt content of the HLW surrogate solution that was obtained under neutralization was 369.5 g·L^−1^, and filling of the compound by the salts of neutralized surrogate solution was 12.8 wt %, and the specific activity of ^239^Pu and ^241^Am was 1.8 × 10^5^ and 2.4 × 10^4^ Bq·g^−1^ (hereinafter, compound #3). The content of the components of the MKP compounds is presented in Table 2.

**Figure 1:**
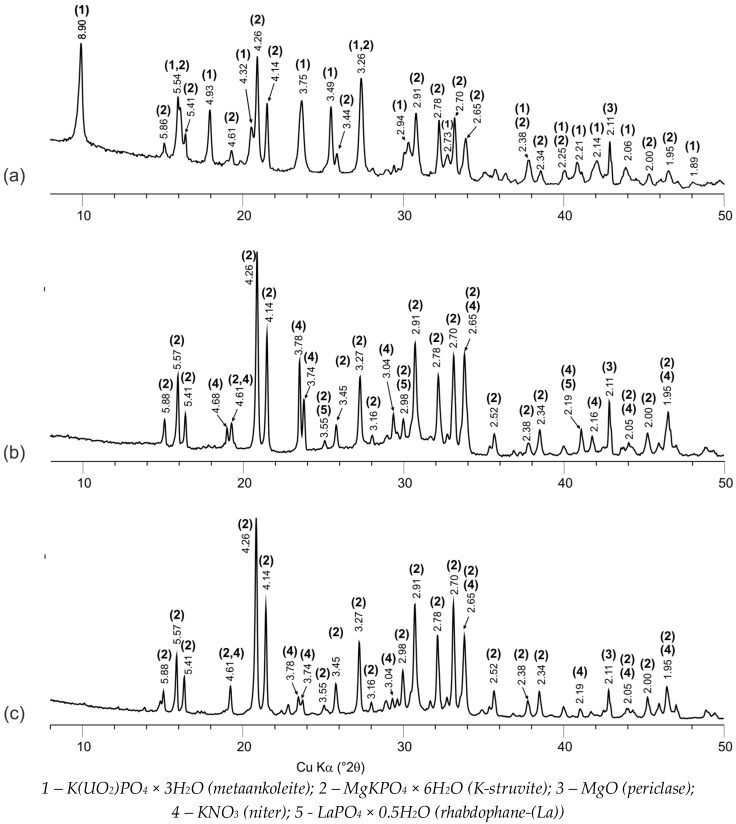
X-ray diffraction patterns of the compounds: #1 (**a**) and #2 (**b**), containing 6.2 wt % and 6.7 wt % uranium and lanthanum, respectively, and #3 (**c**), obtained by solidification of HLW surrogate solution.

**Figure 2:**
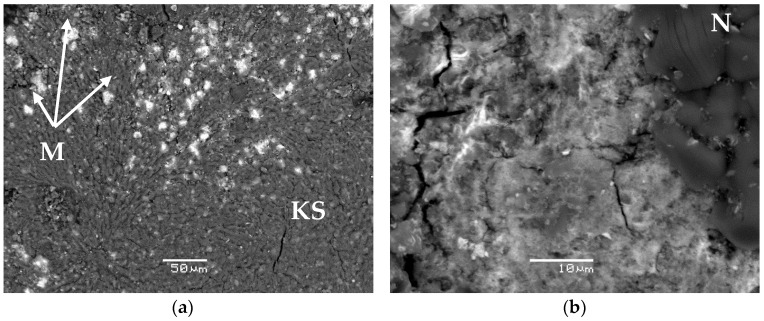

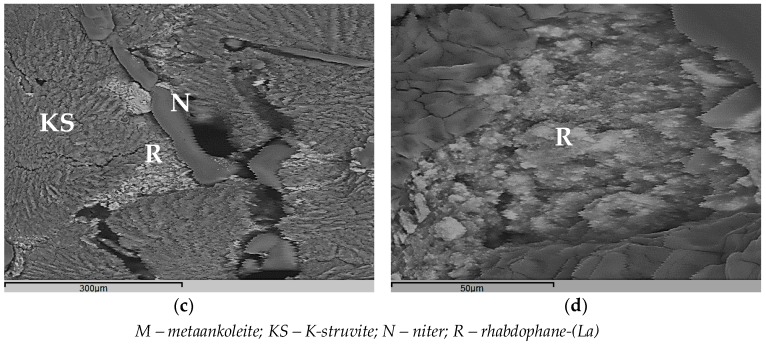
Scanning electron microscopy (SEM) images of the compounds #1 (**a**,**b**) and #2 (**c**,**d**), containing 6.2 wt % and 6.7 wt % uranium and lanthanum, respectively.

The obtained data on the study of the phase composition and structure of synthesized compounds #1–3 by XRD and SEM methods are shown in Figure 1 and Figure 2, respectively.

**Figure 3:**
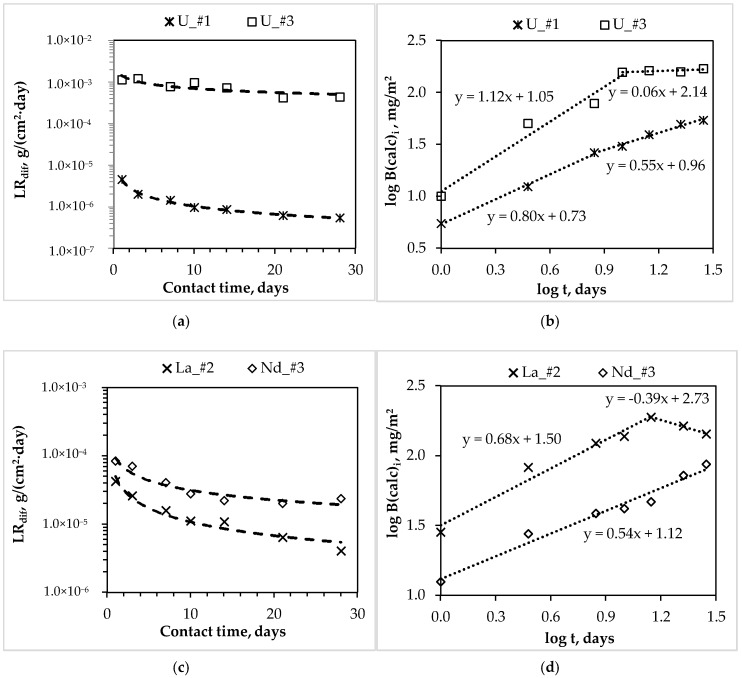

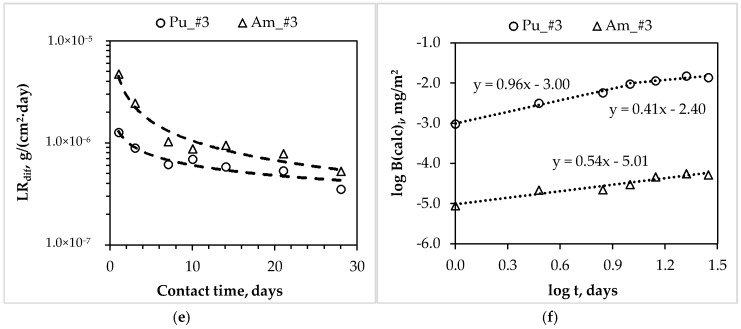
Dependence of the differential leaching rate (LR_dif_) of actinides and rare earth elements (REE) (**a**,**c**,**e**) and the logarithmic dependence of their yield (log B (calc)_i_) from compounds (**b**,**d**,**f**) on contact time with water (indices #1, 2, 3 correspond to compounds containing actinides and REE).

In accordance with GOST R 52126-2003 [18], the differential leaching rates of actinides and REE from synthesized compound #1–3 during 28 days of contact with water (Figure 3a,c,e) were determined, and the mechanisms of their leaching (Figure 3b,d,f, as summarized in Table 3) were estimated.

## 4. Discussion

### 4.1. Phase Composition and Structure of MKP Compounds

The base of all the studied samples of compounds is the crystalline phosphate phase—the synthetic analogue of the K-struvite natural mineral MgKPO_4_ × 6H_2_O (the characteristic peaks at 4.26, 4.14, 2.91, 2.70 Å) (Figure 1a–c). In this case, compound #1 also contains a significant amount of the hydrated potassium uranyl orthophosphate phase, the X-ray diffraction parameters, of which correspond to the metaankoleite mineral K(UO_2_)PO_4_ × 3.0H_2_O (the characteristic peaks at 8.90, 3.75, 3.49 Å) (Figure 1a). This is confirmed by the results of calculating the compound #1 phase composition, according to the microanalysis data: the uranium-enriched particles (denoted by M in Figure 2a) contain up to 45 wt % of uranium and have an average composition Mg_0.33_K(UO_2_)_0.67_PO_4_ × 4.0H_2_O, which corresponds to a mixture of metaankoleite K(UO_2_)PO_4_ × 3.0H_2_O and the K-struvite MgKPO_4_ × 6.0H_2_O in a molar ratio of 2/1. In this case, the main phase of compound #1 (phase KS in Figure 2a) contains up to 3 wt % of uranium.

As a result of potassium replacement with metals of nitric acid solutions, the KNO_3_ (niter) phase (the characteristic peaks at 3.78, 3.74, 3.04 Å) is formed in the obtained compounds (Figure 1), which were also shown previously in [9,10]. However, it was not possible to identify this phase in compound #1 (Figure 1a) by XRD method, probably because of its small content (theoretically not more than 5 wt %), but its presence is clearly confirmed by microanalysis data (phase N in Figure 2b). Impurities of magnesium, phosphorus, and uranium in phase N (Figure 2b) do not exceed 0.6, 1.4, and 1.3 wt %, respectively.

According to XRD (Figure 1b) and SEM (R in Figure 2c,d), it was established that lanthanum as REE in compound #2 is present as a phosphate compound of the analogue of the natural mineral rhabdophane—(La) LaPO_4_ × 0.5H_2_O. In this case, according to the microanalysis data, the main phase of compound #2 (phase KS Figure 2c) is a phosphate compound of the composition Mg_0.60_K_0.68_La_0.36_PO_4_ × 6.3H_2_O, similar to K-struvite. In compound #2, the presence of the KNO_3_ phase (N in Figure 2c) is also clearly confirmed by SEM data.

The MgO (periclase) phase (the characteristic peak at 2.11 Å) is present in all of the studied compounds (Figure 1), and it is associated with an excess of the 10 wt % used MgO relative to the stoichiometry of the reaction (1) in accordance with the technique [10].

### 4.2. The Leaching Rate and Mechanism of Actinides and REE from MKP Compounds

The leaching rate of all metals decreases depending on the contact time of the studied compounds with water (Figure 3a,c,e). However, a significant difference in the rate of uranium leaching from compounds #1 and #3 was established: At the 28th day, the differential uranium leaching rate is 5.5 × 10^−7^ and 4.4 × 10^−4^ g/(cm^2^·day), respectively (Figure 3a). It was determined by XRD and SEM (Figure 1a and Figure 2a) that uranium in compound #1 is bound in a slightly soluble phosphate, which is analog of the natural mineral metaankoleite [24,25], which provides a high resistance of compound #1 to uranium leaching. Obviously, the formation of such stable phase did not occur under the high-salt HLW surrogate solution solidification in compound #3, due to the presence of a large amount of nitrates of various metals (salt background—369.5 g·L^−1^).

Data on the uranium leaching mechanism from compounds #1 and #3 (Figure 3b, Table 3) confirm the difference in leaching behavior. The logarithmic dependence of the uranium yield from compound #1 on the time of contact with water can be divided into two sections, which are described by linear equations with the slopes are 0.80 and 0.55 for seven days from the beginning of the test and the next 21 days, respectively. Thus, the uranium leaching mechanism varies depending on the duration of contact of the compound with water. So, during the first seven days, uranium leaching occurs due to surface dissolution of the compound, where individual particles of hydrated uranyl nitrate were localized. In the next 21 days, the uranium leaching is precisely determined by the diffusion transport from the inner layers of the compound. The uranium leaching from compound #3 in the first 10 days, is determined by the intensive surface dissolution, which is probably enriched by uranyl nitrate, and in the next 18 days—due to the gradual surface depletion (the slopes are 1.12 and 0.06, respectively).

The leaching rate of trivalent REE from the MKP compound increases with the increasing of content of various salts in the compound (for example, lanthanum and neodymium, Figure 3c). So, for compounds #2 and #3 the differential leaching rate of REE on the 28th day is 4.1 × 10^−6^ g/(cm^2^∙day) for lanthanum from compound #2, and 2.4 × 10^−5^ g/(cm^2^∙day) for neodymium from compound #3. It is obvious (Figure 3d) that, under the contact of compound #2 with water, the lanthanum leaching rate will decrease, since lanthanum leaching for 14 days is probably due to the surface dissolution (slope = 0.68) of soluble lanthanum forms of compound #2 in consequence of significant its content in the compound (6.2 wt %), and after 14 days by surface depletion (slope = −0.39). It is important to note that the neodymium leaching from compound #3 is uniquely determined by diffusion from the inner layers of the compound (slope = 0.54), which probably contains a uniformly distributed phase of hydrated neodymium nitrate that is unbound in slow-soluble phosphate forms.

The ^239^Pu leaching rate is the main criterion of matrix quality evaluation for HLW immobilization. It has been established that compound #3 reliably kept both plutonium and americium: the differential leaching rate of ^239^Pu and ^241^Am on the 28th day is 3.5 × 10^−7^ and 5.3 × 10^−7^ g/(cm^2^∙day), respectively (Figure 3e). The plutonium yield from compound #3 in the leaching agent in the first 10 days occurred under the surface dissolution of the compound (slope = 0.96), and then by diffusion transport (slope = 0.41, Figure 3f). Americium leaching is also determined by diffusion transport (slope = 0.54), which is probably from the slow-soluble mixed orthophosphate (Am, REE)PO_4_, which is an analogue of the natural mineral monazite.

The established low value of the ^239^Pu leaching rate from MKP compound is close to the standard requirements for the glass-like compound for HLW immobilization (1 × 10^−7^ g/(cm^2^∙day)). However, it is important to note that MKP compound is synthesized at room temperature, whereas vitrification requires the use of expensive high-temperature electric furnaces or special melters, the liquidation of which after the end of the service life is a great radioecological problem, which is yet unsolved. Thus, the MKP compound approbation for immobilization of real wastes samples that were obtained by radiochemical plants during reprocessing of SNF, and a systematic comparison of MKP and glass-like compound quality indicators, including taking into account the technical and the economic evaluation of these technologies, are of scientific interest.

## 5. Conclusions

As a result of the research, it was established that MKP compounds that were synthesized at room temperature under solidification of nitric acid solutions, which are the surrogate solution of LRW, having complex chemical composition and containing actinides and REE, consisting of crystalline phases—analogues of natural phosphate minerals and possess high hydrolytic stability. Thus, the MKP compound is promising for the immobilization of LRW and it can be an alternative material combining the advantages of technology implementation simplicity that is similar to cementation and high physical and chemical stability corresponding to the glass-like compound.

## Figures and Tables

**Table 1:** Characteristics of high level waste (HLW) surrogate solution.

Specific Activity of Actinides (Bq·L^−1^)	Metal Content (g·L^−1^)	HNO_3_ Content (mol·L^−1^)	Density (g·L^−1^)	Salt Content (g·L^−1^)
^239^Pu – 3.8 × 10^8^ ^241^Am – 5.2 × 10^7^	Na – 13.3; Sr – 3.9; Zr – 7.6; Mo – 0.9; Pd – 5.4; Cs – 9.3; Ba – 6.4; Nd – 28.8; Fe – 1.0; Cr – 2.8; Ni – 0.5; U – 3.1	3.2	1210	206.6

**Table 2:** Composition of magnesium potassium phosphate (MKP) compounds under study.

Compound	Liquid Waste (wt %)	Binders (wt %)		
		KH_2_PO_4_	H_3_BO_3_	MgO
#1	39.5	44.2	1.5	14.8
#2	43.4	41.3	1.5	13.8
#3	41.5	42.9	1.3	14.3

**Table 3:** The leaching mechanism of components of the MKP compounds (indices #1, 2, 3 correspond to the names of compounds containing actinides and REE).

Components of the MKP Compounds	Correspond Figure	Contact Time of the Samples with Water, Days	Slope of the Lines	Leaching Mechanism
U_#1	3b	1–7 7–28	0.80 0.55	dissolution diffusion
U_#3	3b	1–10 10–28	1.12 0.06	dissolution depletion
La_#2	3d	1–14 14–28	0.68 −0.39	dissolution depletion
Nd_#3	3d	1–28	0.54	diffusion
Am_#3	3f	1–28	0.54	diffusion
Pu_#3	3f	1–10 10–28	0.96 0.41	dissolution diffusion
